# Study of Chronic Diseases (Edoc): methodological aspects

**DOI:** 10.11606/S1518-8787.2019053000847

**Published:** 2019-01-18

**Authors:** Thatiana Lameira Maciel Amaral, Cledir de Araújo Amaral, Margareth Crisóstomo Portela, Gina Torres Rego Monteiro, Maurício Teixeira Leite de Vasconcellos

**Affiliations:** I Universidade Federal do Acre. Centro de Ciências da Saúde e do Desporto. Programa de Pós-Graduação em Saúde Coletiva. Rio Branco, AC, Brasil; II Instituto Federal de Educação, Ciência e Tecnologia do Acre. Campus Rio Branco. Rio Branco, AC, Brasil; III Fundação Oswaldo Cruz. Escola Nacional de Saúde Pública Sérgio Arouca. Rio de Janeiro, RJ, Brasil; IV Fundação Instituto Brasileiro de Geografia e Estatística. Escola Nacional de Ciências Estatísticas. Rio de Janeiro, RJ, Brasil

**Keywords:** Adult, Aged, Chronic Disease, Epidemiology, Health Surveys, Methods, Adulto, Idoso, Doença Crônica, Epidemiologia, Inquéritos Epidemiológicos, Métodos

## Abstract

**OBJECTIVE:**

Describe the sampling design and other methodological aspects of the Study of Chronic Diseases (Edoc).

**METHODS:**

Edoc comprises two household surveys with distinct populations, one with adults aged 18 to 59 years (Edoc-A) and another with older adults aged 60 years or more (Edoc-I), living in Rio Branco, Acre. The selection of the participants used complex samples by clusters in two stages of selection, census enumeration areas (CEA) and household. In the first stage, common to both surveys, 40 CEAs were selected with probability proportional to size, and in the second stage, independent for each survey, households were selected with equal probability, and all the residents eligible for each survey were selected. Sampling weights were estimated by the inverse of the product of inclusion probabilities at each stage and then calibrated to produce unbiased population estimates. Interviews were held with questionnaires about socioeconomic and demographic conditions, life habits and health conditions. Anthropometric measures focused on measures of body height, girths and mass, while the vital signs analyzed were blood pressure, heart rate and respiratory rate. Blood and urine samples were collected for analysis.

**RESULTS:**

The Edoc comprised 1,701 participants, 685 of Edoc-A and 1,016 of Edoc-I. Considering the loss of information of some participants and the need of studying specific themes with production of population inferences, 16 subsamples of complete information by theme were generated and two subsamples were exclusive of Edoc-I.

**CONCLUSIONS:**

The Edoc has as important developments the analyses of epidemiological profile of the population from the capital of the state of Acre, contributing to the production of knowledge in public health with useful information for decisions in public health policies.

## RESUMO

**OBJETIVO:**

Descrever o desenho amostral e os demais aspectos metodológicos do Estudo das Doenças Crônicas (Edoc).

**MÉTODOS:**

O Edoc compreende duas pesquisas domiciliares com populações distintas, uma com adultos de 18 a 59 anos (Edoc-A) e outra com idosos de 60 anos ou mais (Edoc-I), residentes em Rio Branco, Acre. A seleção dos participantes usou amostras complexas por conglomerados em dois estágios de seleção, setor censitário e domicílio. No primeiro estágio, comum às duas pesquisas, foram selecionados 40 setores censitários com probabilidade proporcional ao tamanho, e no segundo estágio, independente para cada pesquisa, foram selecionados os domicílios com equiprobabilidade, sendo todos os residentes elegíveis para cada pesquisa selecionados. Os pesos amostrais foram calculados pelo inverso do produto das probabilidades de inclusão em cada estágio e posteriormente calibrados para produzir inferências populacionais. Foram realizadas entrevistas com questionários sobre condições socioeconômicas e demográficas, hábitos de vida e condições de saúde. Mensurações antropométricas centraram-se em medidas de estatura, massa e perímetros corporais, enquanto os sinais vitais analisados foram pressão sanguínea, frequência cardíaca e frequência respiratória. Amostras de sangue e urina foram coletadas para análise em laboratório especializado.

**RESULTADOS:**

O Edoc compreendeu 1.701 participantes, 685 do Edoc-A e 1.016 do Edoc-I. Considerando a perda de informação pontual de alguns participantes e a necessidade do estudo de temas específicos com produção de inferências populacionais, foram realizadas 16 subamostras de informações completas por tema de análise, sendo duas exclusivas do Edoc-I.

**CONCLUSÕES:**

O Edoc tem como desdobramentos importantes as análises do perfil epidemiológico da população da capital do estado do Acre, contribuindo para a produção de conhecimentos em saúde coletiva com informações úteis para decisões em políticas públicas de saúde.

## INTRODUCTION

This article aims to document the methods applied in the Study of Chronic Diseases (Edoc) in Rio Branco, state of Acre. This study is composed of two household surveys: one with adults, named Edoc-A, and another with older adults, Edoc-I.

Although distinct and independent, these two surveys shared some objectives and methods. This survey aimed to analyze prevalence and factors associated with health problems in adults and older people from Rio Branco, state of Acre. To do so, data about the characteristics of the household were collected in both surveys, such as socioeconomic and demographic conditions, exposure to substances and heavy metals, food and life habits, health conditions. Anthropometry was conducted, and biological material was collected. In the specific case of Edoc-I, data about the functional autonomy and depression were obtained. The samples were collected and selected in two stages (census enumeration area and household), with the first selection stage being common to both surveys.

Another important point is that both surveys have distinct and mutually exclusive populations, which allows estimating data for the union of populations and shaping the data of both surveys in an integrated manner.

This study describes the survey populations, sampling design, instruments used, training and pilot test, procedure of data collection, data entry and information consistency editing, in addition to setting up the database and the files by theme.

The project regarding both surveys was approved by the Research Ethics Committee of the Federal University of Acre, under the Certificate of Ethical Assessment (CAAE) 17543013.0.0000.5010, and all the participants signed the informed consent form.

### Survey Population

The city of Rio Branco is crossed by the Acre River, which gives its name to the state and divides it into two districts. On July 31, 2010, the state had a population of 336,038 inhabitants, 96,276 households and 338 census enumeration areas (CEAs), which were defined by IBGE for 2010 Population Census (CD2010). Rio Branco was the sixth largest city in the North region^1^.

The survey population of Edoc-A comprises the residents aged 18 to 59 years, while Edoc-I comprises the residents aged 60 years or more. According to CD2010, Rio Branco had 204,094 adult inhabitants and 14,480 older people. Individuals with compromises that hindered communication or the understanding of the questions were excluded from both research populations, as well as pregnant women.

Thus, the union of both surveys defines the total survey population of Edoc, i.e. the set of residents aged 18 years or more in the city of Rio Branco.

### Sampling Design

Both surveys used cluster sampling plans in two stages. In the first stage, CEAs with probability proportional to the number of households observed in CD2010 were selected. In the second stage, the households were selected systematically with equal probability for each survey.

According to Cochran^2^, by assuming simple random sampling without replacement (SRS), the sample size (n_AAS_) needed to estimate a prevalence P with absolute error d and confidence level 1-α is given by:

$$n_{\text{AAS}}=\frac{Z_{\text{α}/2}^{2}P(1-\text{P)}}{d_{2}}$$

However, the research did not use SRS, but the sampling plan described earlier. To consider the effects of this sampling design in sample size, Pessoa and Silva^3^ recommend multiply the sample size obtained by the above expression for an estimate of the design effect (Deff) of the variable used in sample size determination.

The sample size of Edoc-A was determined by assuming a prevalence of 15% in kidney function alteration among adults (18 to 59 years)^4^, with 95% confidence interval and 3% absolute error. The application of these parameters has led to an SRS of 278 adults. However, previous research does not contain data of Deff for kidney function alteration. As it was preferable to fix an arbitrary value of Deff instead of making no sample size adjustment for cluster effects, a Deff value of 1.95% was used, which resulted in a sample of 543 adults.

The sample size of Edoc-I was estimated using a prevalence of 40% in kidney function alteration among older adults^5^, keeping the other parameters, which resulted in a sample of 1,020 people.

Before determining the number of households and CEAs to be selected, the size of the samples were increased to compensate for possible nonresponses, assuming 20% of loss for adults and 12.5% for older adults, who are more easily found in households and tend to refuse to participate less than adults. Thus, samples of adults and seniors went from 652 and 1,148, respectively, totaling 1,800 people being interviewed in both surveys.

Then, using the mean of adults (1.482) and older people per household (0.394) in the city, we noted that it was necessary to select 440 and 2,914 households to achieve the sample sizes for adults and seniors, respectively. Once such numbers were fixed, we defined the selection of 40 CEAs, the same for both surveys.

The CEAs were selected with probability proportional to size (PPS), to deal with different in cluster sizes. Eleven households were selected, by systematic sampling, for Edoc-A and 73 households for Edoc-I, independently, with interval selection equal to HH_i_/11 and HH_i_/73, where HH_i_ is the number of households in CEA i. Thus, although the household selections are independent, a same household could be selected for both surveys. In the selected households, all eligible residents were interviewed. Therefore, there was no selection of residents in households. The Figure presents the probabilistic schemas of samples from both surveys.

**Figure:**
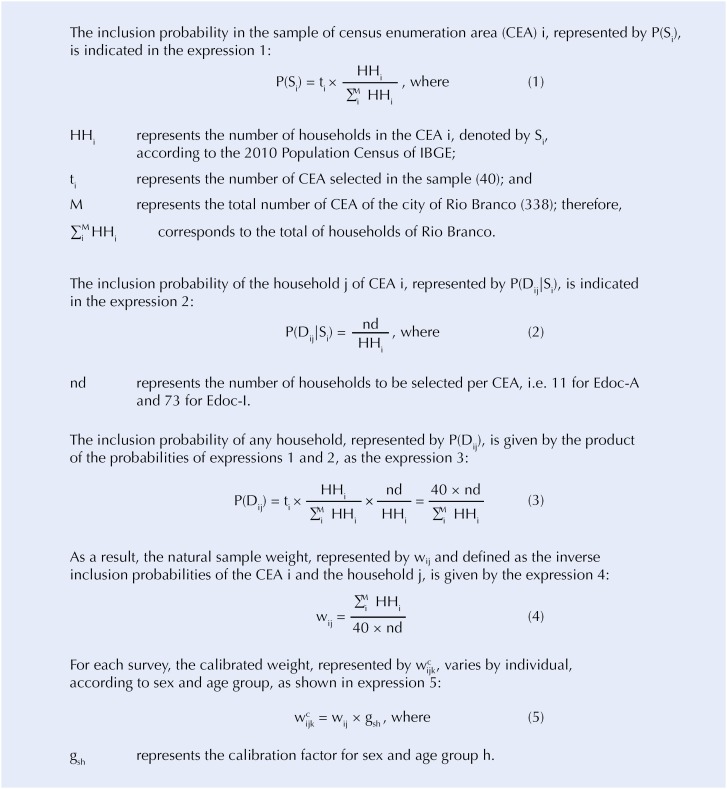
Probabilistic schema of Edoc-A and Edoc-I samples. Edoc-A: Study of Chronic Diseases with adults; Edoc-I: version with older adults

The Figure shows that the sample of each survey is self-weighted (i.e. it has constant weight), since the natural weight of the household is constant in each survey, i.e. it corresponds to 96,276 / (11 × 40) = 218.809090909091 for Edoc-A and 96,276 / (73 × 40) = 32.9712328767123 for Edoc-I.

However, as occurs in all household surveys, there may be nonresponses and biases in the distribution of population by sex and age. The solution usually adopted has been the calibration of sampling weights^6^. This solution became popular because the estimator that results from the use of calibrated weights is equivalent to the generalized regression estimator that considers the same variables used in calibration as independent variables. This implies that the calibration takes advantage of a model that relates the variables of interest with the set of auxiliary variables considered in calibration. Through this model, we seek to reduce the variance of the estimator and, in cases where differential nonresponse occurs, to reduce a possible bias.

The variables used in the calibration were sex and age group (18 to 29 years, 30 to 39 years, 40 to 49 years, 50 to 59 years, 60 to 69 years, 70 to 79 years and 80 years or more). The population totals used in the calibration of weights by sex and age group was estimated for July 1, 2014 (central date of the data collection), using the linear trend method^7^, the same used by IBGE in its population projections. All CEAs selected were interviewed during the period from April to September 2014.

Table 1 presents the projected data, as well as the estimates obtained with the initial weight (inverse inclusion probabilities) and calibrated weight. The table shows that the effective samples had 685 adults and 1,016 older adults, despite the occurrences of nonresponse common in household surveys.

**Table 1:** Resident population on July 1, 2014, in Rio Branco, state of Acre, effective sample size, estimates and relative error, by type of sample weight used, according to the survey, sex and age group.

Survey, sex and age group			Population projection (July 1, 2014)	Effective sample size	Initial weight		Calibrated weight	
					Estimate	Relative error (%)	Estimate	Relative error (%)
Edoc			235,318	1,701	183,381	-22.07	235,318	0.00
	Women		122,798	1,071	123,212	0.34	122,798	0.00
	Men		112,520	630	60,169	-46.53	112,520	0.00
Edoc-A			211,902	685	149,883	-29.27	211,902	0.00
	Women		110,278	473	103,496	-6.15	110,278	0.00
		18 to 29 years	45,290	160	35,009	-22.70	45,290	0.00
		30 to 39 years	30,714	103	22,537	-26.62	30,714	0.00
		40 to 49 years	20,983	100	21,881	4.28	20,983	0.00
		50 to 59 years	13,291	110	24,069	81.09	13,291	0.00
	Men		101,624	212	46,387	-54.35	101,624	0.00
		18 to 29 years	42,443	57	12,472	-70.61	42,443	0.00
		30 to 39 years	28,000	56	12,253	-56.24	28,000	0.00
		40 to 49 years	19,059	45	9,846	-48.34	19,059	0.00
		50 to 59 years	12,122	54	11,816	-2.52	12,122	0.00
Edoc-I			23,416	1,016	33,498	43.06	23,416	0.00
	Women		12,520	598	19,716	57.48	12,520	0.00
		60 to 69 years	7,118	301	9,924	39.42	7,118	0.00
		70 to 79 years	3,611	200	6,594	82.61	3,611	0.00
		80 years or more	1,791	97	3,198	78.56	1,791	0.00
	Men		10,896	418	13,782	26.49	10,896	0.00
		60 to 69 years	6,276	194	6,396	1.91	6,276	0.00
		70 to 79 years	3,076	147	4,847	57.57	3,076	0.00
		80 years or more	1,544	77	2,539	64.44	1,544	0.00

Edoc: Study of Chronic Diseases; Edoc-A: version with adults; Edoc-I: version with older adults Relative error = (estimate – population) × 100/population.

The comparison of sizes of both samples demonstrates that the loss rate of 20% for the sample of Edoc-A was overestimated (685 interviews were obtained, against a forecast of 543), while the loss rate for Edoc-I was well estimated (1,016 interviews were obtained, against a forecast of 1,020). Additionally, the initial weight leads to an underestimate of 22.1% of the population size, composed by an underestimate of 29.3% for adults and by an overestimate of 43.1% for older adults. In addition, the analysis by sex confirms what is known in sampling as availability bias: women tend to be more available at home to be interviewed, both among adults and older people.

However, it was not always possible to obtain all the information about everyone. Thus, nonresponses were observed in different degrees according to the theme. A possibility to handle that would be to adopt a mechanism of probabilistic imputation, which would generate a file with all the information. However, considering the sampling size and the difficulty of ascribing results of laboratory tests or physical assessment, we decided to give a treatment similar to that adopted in the Study of Cardiovascular Risks in Adolescents (Erica), which designed different databases with the variables required for each analysis theme and the set of records with the complete information. Thus, the calibration of weights in each thematic database was used to treat nonresponses and correct possible biases in the calibration variables^8^.

Seventeen databases were created, one for the set of interviews (which includes specific nonresponses) and 16 for the subsamples of complete information about each theme. For Edoc-A, fourteen files of complete subsamples were created, which were given the following names: albumin, alcohol, waist circumference, cholesterol, creatinine, diabetes, dyslipidemia, hand grip strength, hemoglobin, hypertension, obesity, metabolic syndrome, smoking and triglycerides. In the case of Edoc-I, two other themes were the subject of exclusive research: functional autonomy and depression.

Tables 2 and 3 indicate that there have been few nonresponses per theme. Yet, in all these files, the nonresponse received the same treatment of calibration of weights described earlier for the set of interviews. Therefore, all estimates coincide with the population totals listed in Table 1.

**Table 2:** Size of the effective sample by subsample for anthropometric data, life habits and health conditions, according to the survey, sex and age group.

Survey, sex and age group			Total	Alcohol	WC	HGS	SAP	Obes	Smoking	FA	Depr
Edoc			1,701	1,631	1,612	1,609	1,638	1,598	1,695	1,011	1,008
	Women		1,071	1,034	1,017	1,015	1,035	1,010	1,067	595	595
	Men		630	597	595	594	603	588	628	416	413
Edoc-A			685	659	645	643	644	641	679	–	–
	Women		473	456	447	446	447	445	469	–	–
		18 to 29 years	160	151	147	146	144	146	158	–	–
		30 to 39 years	103	103	96	96	96	97	102	–	–
		40 to 49 years	100	95	98	98	100	97	99	–	–
		50 to 59 years	110	107	106	106	107	105	110	–	–
	Men		212	203	198	197	197	196	210	–	–
		18 to 29 years	57	55	51	51	49	49	56	–	–
		30 to 39 years	56	52	49	49	50	49	56	–	–
		40 to 49 years	45	45	44	44	44	44	45	–	–
		50 to 59 years	54	51	54	53	54	54	53	–	–
Edoc-I			1,016	972	967	966	994	957	1,016	1,011	1,008
	Women		598	578	570	569	588	565	598	595	595
		60 to 69 years	301	292	286	285	292	284	301	299	299
		70 to 79 years	200	192	191	191	200	188	200	199	199
		80 years or more	97	94	93	93	96	93	97	97	97
	Men		418	394	397	397	406	392	418	416	413
		60 to 69 years	194	185	182	183	186	181	194	194	192
		70 to 79 years	147	138	142	140	144	138	147	146	145
		80 years or more	77	71	73	74	76	73	77	76	76

Edoc: Study of Chronic Diseases; Edoc-A: version with adults; Edoc-I: version with older adults; Total: all interviews; WC: waist circumference; HGS: hand grip strength; SAP: systemic arterial pressure; Obes: obesity; FA: functional autonomy; Depr: depression symptoms and signs

**Table 3:** Size of the effective sample by subsample for data from laboratory results and morbidities evaluated, according to the survey, sex and age group.

Survey, sex and age group			Total	Alb	TC	Sc	DM	DLP	Hb	MS
Edoc			1,701	1,615	1,631	1,632	1,637	1,632	1,626	1,617
	Women		1,071	1,018	1.029	1.029	1,035	1,030	1,026	1,018
	Men		630	597	602	603	602	602	600	599
Edoc-A			685	637	649	649	651	649	649	642
	Women		473	441	451	451	453	451	451	446
		18 to 29 years	160	146	150	150	150	150	150	146
		30 to 39 years	103	92	97	97	98	97	97	97
		40 to 49 years	100	98	98	98	98	98	98	98
		50 to 59 years	110	105	106	106	107	106	106	105
	Men		212	196	198	198	198	198	198	196
		18 to 29 years	57	51	51	51	51	51	51	50
		30 to 39 years	56	49	50	50	50	50	50	49
		40 to 49 years	45	44	44	44	44	44	44	44
		50 to 59 years	54	52	53	53	53	53	53	53
Edoc-I			1,016	978	982	983	986	983	977	975
	Women		598	577	578	578	582	579	575	572
		60 to 69 years	301	288	289	289	291	290	287	288
		70 to 79 years	200	196	195	195	196	195	194	192
		80 years or more	97	93	94	94	95	94	94	92
	Men		418	401	404	405	404	404	402	403
		60 to 69 years	194	183	185	186	185	185	185	184
		70 to 79 years	147	143	144	144	144	144	142	144
		80 years or more	77	75	75	75	75	75	75	75

Edoc: Study of Chronic Diseases; Edoc-A: version with adults; Edoc-I: version with older adults; Total: all interviews; Alb: albumin; TC: total cholesterol; Sc: serum creatinine; DM: Diabetes mellitus; DLP: Dyslipidemia; Hb: hemoglobin; MS: metabolic syndrome

### Instruments: Questionnaires and Measurement Protocols

Three semi-structured questionnaires were used for the interviews: 1) household, containing information of family, housing, sanitation and the Socioeconomic Classification^9^; 2) individual, specific for adults; and 3) individual, specific for older people. The last two were structured in thematic modules with socioeconomic, demographic and information about occupational exposures, life habits, health and quality of life. The questionnaire for older adults had modules relating to functional autonomy and depression.

The socioeconomic and demographic information included data on place of birth, age, sex, skin color or ethnicity, educational level, marital status, work and occupation. Occupational exposure data included information on toxic agents, route and exposure time.

Data on life habits, such as smoking and alcoholism, were the report of daily and occasional consumption, performed before the survey. To collect information on physical activity while commuting to work or school, at work, at home and during leisure time, we used the questions employed in the study of Surveillance System for Risk and Protective Factors for Chronic Diseases by Telephone Survey in 2013^10^, which deals with the type, frequency and duration of these activities.

Still in the module of behavioral information, we used the Quantitative Food Frequency Questionnaire, previously validated^11^, which is considered an important tool for assessing the dietary intake in epidemiological studies^12^.

In the module about health, the questions dealt with history of first-degree relatives for cardiovascular, renal and metabolic morbidities. Questions about self-assessment of health, self-reported morbidities, pain and complaints of the physical state and about the perception of stress were also included. Information about the current use of medication, dose and frequency, through presentation of a prescription or packaging of the product was also obtained, as well as long-term use, for one month or more, of antihypertensive medications, insulin, anti-diabetic medication, antibiotics, non-steroidal anti-inflammatory medications, antidepressant/anti-anxiety drugs and immunosuppressants. The use and evaluation of health services were also investigated.

To assess the quality of life, the quality of life questionnaire of the World Health Organization (WHO) was applied, the WHOQOL-Bref^13^.

Additionally, to investigate the functional autonomy among older adults, the Activities of Daily Living scale (ADL) was employed, modified by Katz^14^, and adapted to Brazilian Portuguese^15^, and the scale of Instrumental Activities of Daily Living (IADL)^16^. To screen the presence of depression in older adults, the Geriatric Depression Scale (GDS-15) was employed^17^.

The anthropometric data included the measurement of weight, height and waist circumferences, hip, arm and calf, following the protocols recommended by the American College of Sports Medicine (ACSM)^18^, all in duplicate; the averages of the measurements were considered.

The body mass index (BMI) was estimated by dividing the weight (kg) by the square of height (m^2^), subsequently categorized, as well as the waist circumference, according to WHO criteria^19^. The waist-hip ratio (WHR) was also considered, taking into account ACSM cut-points (2006)^18^. Hand Grip Strength (HGS), in kgf, was measured with a hydraulic hand dynamometer, in accordance with the procedures adopted by the American Society of Hand Therapists^20^.

The vital signs data consisted of measuring heart and respiratory rate and blood pressure (BP), determined in accordance with the protocol recommended by the Brazilian Society of Cardiology^21^. The value considered of BP was the average between the second and third measures.

The biological material used included blood and urine samples. The blood samples were obtained by peripheral blood collection, with prior antisepsis of the cubital fossa of the participants. Part of the sample taken, 4 ml, was packed in vacuum test tube without anticoagulant and centrifuged at 1,500 rpm for 15 minutes. The serum extracted was stored for biochemical dosage of triglycerides, total cholesterol and fractions: high-density lipoprotein (HDL), low-density lipoprotein (LDL) and very low-density lipoprotein (VLDL).

Total cholesterol was dosed by colorimetric enzymatic method COD/PAD, in the same way as fractions (HDL, LDL and VLDL) and triglycerides GPO/PAP (Labtest Diagnóstica). LDL was obtained from VLDL by hydrolysis of different lipolytic enzymes.

Four ml of whole blood was packed in a vacuum tube containing 2 mg/ml of ethylenediaminetetraacetic acid (EDTA) for hematological and serum creatinine analysis. The serum creatinine was dosed by traceable enzymatic method of isotope dilution mass spectrometry (IDMS) in an automatic analyzer (Labmax 240 Premium).

The complete blood count was performed by electronic cell counting. The differential leukocyte count was performed by microscopic analysis of 200 cells in a blood distension stained by the Romanowsky method.

For the analysis of serum glycemia, a 4 ml blood sample was used and stored in vacuum tube containing 2 mg/ml sodium fluoride, being centrifuged before analysis. The dosage used the glucose oxidase method (Labtest Diagnóstica).

For urine samples, approximately 50 ml of the midstream of the first morning urine of each individual were collected. The samples were placed in standard bottles and transported from the collection site to the lab for analysis in controlled temperature. They were processed by physicochemical and microscopic analysis of the sediment. Some of it was centrifuged, and the supernatant was removed for biochemical analysis of albuminuria concentrations.

For the urine test type I (ASE), physical, chemical and microscopic analyses were conducted. The amount of albumin and urinary creatinine was determined by turbidimetric analysis POP Kit Labtest^®^ (Labtest Diagnóstica), reacting with a specific antibody.

The analyses of biological material were performed in the same laboratory to ensure standardization of procedures.

### Training and Pilot Test

All data collection procedures were performed by qualified personnel, trained and supervised by the coordination of the study. The team of interviewers was composed by students or health professionals who participated in a preparatory course promoted by the coordination to understand the role, function and importance of the interviewer in scientific research, in addition to the familiarity, understanding and application of the instruments. To standardize the interviews, a manual for study and activities basis was prepared.

The team responsible for the physical assessment, vital signs and collection of biological samples, named the health team, was composed of health professionals: nurses, physical education professional, nursing and laboratory technicians. All professionals were previously trained in order to understand and standardize the procedures adopted.

A pilot study was conducted in a CEA not included in the sample. During mapping, difficulties in locating addresses were corrected, being added the address information and references to the description and visual presentation of the household. Later, interviews were initiated, in which participants were asked to comment on the difficulties in understanding the questions and assess the interviewer and the health team.

For quality control of information, the interviews were reconducted, as well as the physical assessments in 20 adults and 30 older people assessed in the pilot study.

### Data Collection

The mapping of the selected CEA respected the geographical limits and beginning and end established by IBGE, with the election of households always starting by the right limit of each CEA. With the total number of households in each CEA, a calculation was carried out for setting the number of households to be skipped from the first CEA. The households eligible to interview adults, older people or both were indicated in detailed addresses and image in the spreadsheet used by the team of interviewers.

The definition of household adopted was the place structurally separate and independent that is intended to be a dwelling for one or more people or that is being used as such. We selected only private households characterized as residences where the relationship was dictated by ties of kinship, domestic dependency or by living standards, such as houses, apartments and dwelling units in single-room houses or tenements^1^.

With the address lists, the trained professionals interviewed all of the people eligible for the study that lived there. In closed households or in the absence of eligible resident, the team returned up to three times to the local, in different periods, for data collection; when they were not successful, the loss was characterized, without replacement of households. At the end of the interview, a date for the collection of blood and urine and for the physical assessment was scheduled. At that time, guidelines were given on the procedures for the collection of biological samples, as well as for the physical assessment: 12-hour fast for blood collection; collection of the first morning urine, disregarding the first part of the flow and after personal hygiene, in individual bottle available and identified by the interviewers; and use of light clothes, preferably shorts or skirt and shirt, for the physical assessment.

Data were collected from April to September 2014. Before the assessments, research professionals had to come forward with official identity document with photo. In the previously defined date and time (from Monday to Saturday, in the morning), the team previously trained performed physical assessments and collected blood and urine samples.

The results of laboratory tests and physical assessment, upon examination by authorized health professional, were delivered to the participants. In case of alterations, the research team scheduled the appointment with a medical professional in the health unit of reference to the neighborhood. For the schedule of appointments in public health units, partnerships with health departments in the state of Acre and in the city of Rio Branco were conducted.

### Consistency of Data

The questionnaires and evaluations were reviewed and coded simultaneously to data collection. The information was coded in featured next to the original question, for easier typing, being subsequently held dual independent typing in Microsoft Office Access, with the adoption of filters to avoid typos. The database was compared and corrected using EpiInfo^®^ program, version 3.5.2.

The inconsistencies observed were corrected by reviewing the original instruments (questionnaires, physical assessment and results of laboratory tests), without imputation of data.
